# The Normalization of Conservative Gender Politics in Chile and the Role of Civil Society

**DOI:** 10.3389/fsoc.2020.00017

**Published:** 2020-03-20

**Authors:** Patricia Graf

**Affiliations:** Department for Economic Psychology, BSP Business School Berlin, Berlin, Germany

**Keywords:** transition to democracy, civil society, women's movement, authoritarian gender regime, domestic violence, divorce law

## Abstract

The article discusses two cases of gender policy making during the Chilean transition to democracy, the policy on domestic violence and the divorce law. By comparing the official discourses on these two policy projects we show that authoritarian gender regimes can resist transition to democracy despite a vivid civil society. The case of Chile was selected, on one hand, because it exhibits particularly resistant authoritarian institutional enclaves. On the other hand, Chilean women's movements are often cited as a paragon of women's movements in transitions. Despite the central role of Chilean women's movements as a strong civil society force conservative gender roles and institutions inherited from the autocratic regime (e.g., conservative divorce and reproductive rights) have remained dominant. I argue that during the time of transition conservative political actors, but also parts of civil society, negotiated on these gender roles and institutions and thereby reached a status quo. Recent cases of sexual violence in response to student's uprisings show that this status quo is quite stable and prevents a real coming to terms with state violence.

## Introduction

In October 2019, the prices for metro tickets in the Chilean capital Santiago de Chile increased by 30 pesos. This led to hughe civil protests that are described to be the biggest uprising in the history of the country. The response of the government were massive police and military operations and the declaration of a state emergency. Five homicides, 92 cases of torture and 19 cases of sexual violence commited by state agents—this is the resume the Chilean National Human Rights Institute published regarding the protests (Tapia, 2019).

This resume stands in opposition to the country's model character among Latin American countries. The rule of law and economic performance are considered exemplary (Hillebrand, 2004). Chile is considered a consolidated democracy and an example of successful system transformation (Merkel, 2010). In this article we argue that this paradox picture can be explained by looking at the history of Chilean policy making in the field of gender politics and the role of the Chilean women's movements.

The Chilean women's movements are credited with an important role in this transition to democracy. They are considered as one of the few movements that were allowed to act politically during the military dictatorship and massively protested against human rights violations (Boris, 1998). After the end of the military dictatorship, some protagonists of the women's movement became members of the national women's secretariat, thus shaping the gender politics of the young democracy. Considering the importance of women's movements, it is important to ask why Chile still has numerous disadvantages for women today: It ranks 17th on the Gender Inequality Index^1^. Only 16% of parlamentarians are female, and in 2017 the country was listed as having one of the strictest abortions bans worldwide (Die, 2017).

The case of Chile was selected, on one hand, because it exhibits particularly resistant authoritarian institutional enclaves. On the other hand, Chilean women's movements are often cited as a paragon of women's movements in transitions. In public opinion and scientific literature (Jaquette, 1994; Valenzuela, 1998; Franceschet and Macdonald, 2004), Chilean women's movements are regarded as playing a decisive role in resistance to the dictatorship. Despite the central role of Chilean women's movements as a strong civil society force—especially for gender politics—institutions—and gender images inherited from the autocratic regime (e.g., conservative divorce and reproductive rights) have remained dominant. I argue that in the time of transition there was a “normalization of hegemonic discourses of the authoritarian” (Graf et al., 2017) that stabilized conservative institutions and gender images. In this chapter, I examine the nature of these normalization processes, as well as the role played by civil society in this normalization. The theoretical basis of this chapter is the governmental perspective on gender relations, which assigns an important role to civil society in the stabilization of transition. We will focus on the predominant discourses in democratic opening and closing processes (Graf et al., 2017). This paper will not further deal with the recent cases of sexual violence in Chile. However, we consider the overall framework and the conclusions drawn from the historical cases as relevant in order to explain the recent gender politics.

## Methods

The study of Chilean women's movements is based on a literature review of the role of women's movements in transition and on a secondary analysis of speeches and interviews of movement members and members of the transition regime.

According to Bryman (2015) general search terms were defined (Chile, divorce, sexual violence, women's movement, transition). The search was exercised in Spanish and English on the EBSCO database, the library of the Berlin Ibero American Institute, the Latin American Scielo database, and the library of CEPAL. The literature was selected respective the time frame and the focus on parliamentary and institutional discourses.

From the literature two case studies on two concrete legislative processes, the Law on Domestic Violence and the regulations on marriage and divorce were built. I chose these policies because their analysis clearly demonstrates how the dictatorship's conservative family image was normalized in a democratic context. In a second step, I examine the role of women's movements in democratic transitions. For this purpose, the initial situation of women's movements at the end of the dictatorship is presented. On this basis, I analyze how the women's movement became more and more divided after the end of the dictatorship and how this was accompanied by a normalization of authoritarian discourse. Finally, from the case of Chile, general observations on the normalization of conservative gender discourses in other political systems are drawn.

## Theoretical Background

Gender relations play an important role in securing the stability of authoritarian regimes and in de-democratization. Numerous cases, such as the military dictatorship in Spain or the Peruvian gender policy under Fujimori, highlight the influence of autocratic regimes on gender relations. There are, however, large gaps in research on gender relations in autocracies and transitional regimes. The gender images and roles often found in informal institutions and power relations are neglected due to the common focus on the “deficits” of formal institutions. Studies in comparative analysis have emerged that systematically explore the role of gender relations in autocracies. In recent times, this strand of research has focused on the legitimacy of autocratic regimes (Gerschewski et al., 2013). In this approach, it is assumed that autocratic regimes not only accumulate output legitimacy, for example by means of political results and regime performance, but also generate input legitimacy.

In the following, approaches of citizenship, democratic-theoretical assumptions, processes of norm formation and the approach of gender regimes are used to gender relations in transition processes and to analyze mechanisms for the consolidation of authoritarian gender discourses.

### Citizenship as Status and as Practice

When we talk about processes of democratization, the concept of citizenship usually has the role of pointing out the process of institutionalizing certain rights. However, this conceptualization does not take into account that citizens play an important role in this process. Kabeer (2012) based on Lister (1997) suggests operationalizing the struggle for gender justice as citizenship as practice on the one hand and citizenship as status on the other. With the category of status Kabeer analyzes “how the existing constitutional/legal arrangements in a society define the rights and responsibilities of citizenship, including its gender dimensions”. Practice means “the different ways in which members of a society seek to act and challenge these collective definitions” (Kabeer, 2012, p. 220). The advantage of this dichotomy it the possibility to look at the dissent between actors and the negotiation processes regarding the status of citizenship, i.e., women's rights. This opens a path to the democracy-theoretical debate on citizenship. The concept of citizenship has an ambivalent position in the political science debate. While pluralistic concepts regard the competition of organized interests as the core of democracy (Dahl, 1967), theoretical classics like Hobbes show a mistrust in the citizen (Schmidt, 2013). This ambivalence is also reflected in recent debates on organized civil society. Organized civil society automatically plays the role of the antipode to the autocrats, the role of the “school of democracy” (Graf et al., 2017, p. 73). Feminist research criticizes this perspective as it obscures internal social power processes, for instance between actors of individual movements or between movements and state institutions (Nüthen, 2019). On the one hand, the term “voluntary” seduces to put civil society actors automatically into connection with instruments of deliberation, beyond compulsion and repression. However, there are examples, such as the “Shining Path,” a Peruvian guerrilla movement, which show that social movements exist even without internal democratic processes. On the other hand research on right-wing feminism in Germany (Wielens, 2019) show that social movements and also women's movements do not equated with struggles for democracy. It is therefore important to liberate the concept of citizenship as practice from the normative attitude and to look at the negotiation processes for citizenship as status, i. the concrete struggles for gender justice. How these negotiation processes are related to (de-) democratization processes?

In the following, we will illuminate democratic-theoretical assumptions as well as concepts of authoritarianism research in order to get a better look at the relationship between political regime and citizenship.

### Concepts of Democratization

Democratic regimes achieve input legitimacy through democratic consent. It has long been assumed that among autocratic regimes only the electoral autocracies could access this source of input legitimacy, because they connect the population by means of pseudo-elections (Buzogány et al., 2016). As Hannah Arendt asserted, authoritarian regimes still have other sources of input legitimacy, with ideologies and identity policies playing central roles. Ideology “promises to explain all historical happenings, the total explanation of the past, the total knowledge of the present, and the reliable prediction of the future” (Arendt, 1951/1966, p. 470). Recent reflections on the role of legitimacy in autocracies are taking up this perspective once again (for an overview see Kailitz and Wurster, 2017).

As Arendt pointed out in her remarks on totalitarian rule, totalitarian systems seek to destroy social pluralism: “Ideologies always assume that one idea is sufficient to explain everything in the development from the premise, and no experience can teach anything because everything is comprehended in this consistent process of logical deduction” (Arendt, 1951/1966, p. 471). In terms of gender relations, this means that pluralist gender subject positions are replaced with uniform images. These images—embedded in ideologies and identity policies—are very influential (Wilde, 2012). I argue that this holds true for autocracies, too. Authoritarian regimes can use their gender policies to transport their ideologies and express their visions of family, social security, education, and gender roles (Woods and Frankenberger, 2016).

What is the relationship between the gender policies of autocracies and transitional regimes? Gender research so far has mainly analyzed the effects of gender on formal and informal institutions (Waylen, 2016). The research strand of historical institutionalism (Mahoney and Thelen, 2010) assumes that both formal and informal institutions are not simply replaced but can be “deposited” in later regimes. During regime transitions, powerful actors negotiate which institutions of the authoritarian system will be “inherited” and the limits of civilian rule (Fuentes, 2000). Who are the actors of negotiation? Studies on women's movements in Latin America (Valenzuela, 1998; Franceschet and Macdonald, 2004), authoritarian Spain (Threlfall, 2013), and Eastern Europe (Jaquette and Wolchik, 1998) indicate that some women's movements exerted pressure on authoritarian regimes.

Literature on international norm creation and norm entrepreneurs suggests that organized civil society can contribute to political opening by pressuring the regime to implement international women's rights on a national level (Finnemore and Sikkink, 1998; Rošul-Gaji, 2014). These women's movements successfully linked national and international levels, which enabled them to influence and shape discourse about gender. Knowledge of how discourses are negotiated and shaped is helpful when considering gender in autocracy and transition scholarship. However, as has been pointed out above, feminist research shows that women's movements can also be shaped by internal power struggles and lack of solidarity. This makes it difficult for women's movements to speak with one voice and shape gender discourses.

### Gender Justice as Norm Creation

Analyzing the influence of women's movements on norm creation also requires to analyze the connections between movement actors and the government apparatus. Literature on norm creation is helpful for investigating the breakdown of autocratic discourse, as it emphasizes the dynamics of change and the significance of networked actors in an organized civil society. However, literature on international norm entrepreneurs has a strong normative foundation. In this strand of literature, organized civil society and especially women's movements are perceived to act and to strive only for democratic aims and ways of government. However, by supporting the transition regime, women's movements can also serve to stabilize conservative institutions and gender images. This stabilization is referred to by Graf et al. as a “normalization of antifeminist and conservative gender images” (2017, p. 82): there is the generation of norms, on the one hand, and, on the other, gender images are being explained as “normal,” everyday practice—despite their contestation by parts of civil society. This process of normalization, however, generates input legitimacy for the transition regime because it helps to form a “we-feeling,” or a collective identity.

### Gender Regimes

To refer to such conservative institutions and gender images, I use the term “authoritarian gender regime.” According to Henninger and Ostendorf, gender regimes focus on “the core political question of politics and power” (Henninger and Ostendorf, 2005, p. 20). The focus is thus not only on how formal and informal institutions shape gender relations, but also on the power and dominance relations underlying discursive practices and gender norms (Bothfeld, 2008).

How can we define authoritarian gender regimes? Friedrich and Brzezinski (1965, p. 5)define authoritarianism as “any political system in which the rulers are insufficiently, or not at all, subject to antecedent and enforceable rules of law—enforceable, that is, by other authorities who share in the government and who have sufficient power to compel the lawbreaking rulers to submit to the law.” In the absence of pluralism and diversity, autocracies are based on ideologies and mentalities that support the regime and maintain the unity of national identity (Buzogány et al., 2016). Authoritarian gender regimes are thus characterized by the fact that political positions and resources influencing gender relations are beyond the scope of state institutions and practices^2^. Second, the gender norms and discourses of authoritarian gender regimes are based on ideologies and mentalities that do not allow a variety of gender images. Gender norms that could become “dangerous” for the regime are thus precluded. In transitions, authoritarian gender regimes must compete with democratic gender regimes. The authoritarian gender regime can become normalized in this process.

The mechanisms and discourses contributing to this normalization are investigated in the following in the case of Chilean gender policy during the transitional government of Patricio Aylwin (1989–1994). Subsequently, I analyze the after-effects of gender norms established during the transitional government and how they shaped the scope of reform of gender policy under Michelle Bachelet (2006–2010, 2014–2018)^3^.

## Gender Policies in Chile

Among Latin American countries, Chile is often presented as a model for the successful transition to democracy. Chile is seen as exemplary in terms of it rule of law and economic performance (Hillebrand, 2004). It is categorized as a consolidated democracy and depicted as a successful system transformation (Merkel, 2010). When Patricio Aylwin, the first democratically elected president, took over the political leadership in Chile after 16 years of military rule, he removed the last military dictatorship in the southern part of Latin America. Nevertheless, authoritarian ideologies are still at work in the subjects, mentalities, and institutions and guide the thoughts and actions of the people. Authoritarian enclaves—authoritarian institutional “islands” within a democratic political system—contribute to this effect. These enclaves are still apparent in several policy areas, including the area of gender policy. In the following, I will examine which mechanisms and discourses led to the negotiation and stabilization of these enclaves.

### The Establishment of an Authoritarian Gender Regime During Military Dictatorship

Chilean women's movements played an important part in the transition to democracy. They are considered as one of the few movements capable of acting during the military dictatorship, since civil society organizations operating within the parties and trade unions were violently oppressed (Boris, 1998). The motives of the women who organized during the military dictatorship were highly varied. On the one hand, women of different backgrounds organized soup kitchens in poverty-stricken areas (Boris, 1998). On the other, women increasingly took over the role of breadwinners due to the “disappearance” of many men during the military dictatorship. Also, the neo-liberal, export-oriented policy created new employment opportunities for women, especially in the agricultural sector. Toward the end of the military dictatorship, these women workers organized themselves in the fight for better labor rights (Tinsman, 2000). They also organized to protest human rights violations and to search for those who disappeared.

The military regime was not blind to the formation of women's movements. In an effort to suppress alternative visions of society, eliminate anti-regime tendencies, and control gender relations, Augusto Pinochet transformed the National Women's Secretary, which had been created by President Allende in 1972, into an ideologic propaganda tool. His wife Lucia Hiriart served as director and was joined by several officers' wives and upper-class women in the administration (Chuchryk, 1994). The Women's Secretary was intended to generate legitimacy for the military regime and to propagate Pinochet's vision of the “patriarchal family” as the ideal order. In Pinochet's vision, women were to concentrate on family affairs, patriotic childhood upbringing, welfare, and the fight against poverty (Thomas, 2016). This traditional division (vision) of gender roles was not unique to Pinochet, as it had already been promoted under the rule of Salvador Allende and been institutionalized in the National Women's Secretary (Godoy Ramos, 2013): the slogan of the leading parties under Allende was “Give land to the man who works it,” which institutionalized gender-specific land rights (Tinsman, 2000, p. 158). Fischer describes the Chilean agrarian reform under Allende as representing a “monstrous” masculine, heteronormative ideal (Fischer, 2016, p. 41). To sum up, during the final period of the military dictatorship there was a broad women's movement, which made demands on the military regime. The military regime responded by founding the Secretary as the first Chilean institution for gender policy.

### Normalization of Authoritarian Gender Images During the Transition

In 1988, Pinochet lost his own referendum, which would have secured him a “second term.” This opened the way for Chile's transition to democracy. However, the conservative gender and family images of the dictatorship survived this referendum and were normalized during the 1st years of the transition.

The focus of the first democratically elected transitional government under Patricio Aylwin was how to cope with past human rights abuse. Here, too, conservative gender images were passed down. Since Aylwin was severely restricted and under pressure by numerous prerogatives still possessed by the military, he chose the discourse of the Reconciliación (reconciliation) in order to enforce a human rights policy oriented toward reconciliation and forgiveness. Aylwin applied the strategy of non-cooperation with the military and tried to limit the constitutional power of the military, i.e., the authoritarian enclaves within the democratic constitution. Aylwin's establishing of the Rettig Commission to investigate human rights violations led to the Dia de Enlace: In response to the commission's institutionalization, Pinochet ordered unannounced military exercises throughout the country (Fuentes, 2000). In terms of human rights policy, Aylwin made concessions.

This influenced Aylwin's understanding of the transition, which he claimed was completed with the first democratic election, as well as his assertion that the view should now be directed toward the future (Forstenzer, 2017). Human rights violations should only be pursued “as far as possible” (Hiner and Azocar, 2015). Above all, the Catholic Church was able to make use of its proximity to the Christian Democratic Party in order to implement its vision of reconstruction. Actors of the Church and of the conservative parties were key to shaping the discourse of the Renacimiento, the rebirth of Chile. For this rebirth, a consensus was necessary. Victims of political or sexualized violence as well as of ill-treatment and other criminal acts that did not end with death were excluded from the first report on the crimes of the dictatorship, the so-called Rettig report. Correspondingly, the report produced a picture of a dictatorship that had mainly been violent to men (Hiner and Azocar, 2015). The fact that women during the military dictatorship engaged in acts of political resistance and suffered massive human rights violations is usually excluded from the debates accompanying this development (Hiner and Azocar, 2015). At the presentation of the report, Aylwin reiterated the formula of reconciliation: “For the good of Chile we must look to the future that unites us more than the past that separates us.… Forgiveness requires regret by one party and generosity by the other” (cited in Hiner and Azocar, 2015, p. 57).

The discourse of reconciliation and consensus meant the continuation of the dictatorship's gender policies, namely, they were left untouched or even linked to liberal norms. This continuation was mirrored in the institutional landscape: in 1990 Patricia Aylwin founded the National Women's Service (SERNAM) in continuation of the National Women's Secretary.

The foundation of SERNAM was not without critique, especially from radical women's movements. On the one hand the creation of an institutionalization of women's affairs was a strong claim of the women's movement. On the other hand the relative lack of power of SERNAM was criticized. The influential feminist corporation La Morada, for example, viewed the institutionalization of SERNAM as an important “gesture” (cited in Díaz Rubio, 2012, p. 31) but pointed on the low budget and the lack of the status of a ministry. On the other hand SERNAM's dual responsibility for women's and family affairs was reproved (Díaz Rubio, 2012).

### The Case of Domestic Violence Legislation

This persistence of gender policies is reflected in domestic violence legislation. It was a major concern of feminist organizations in the 1st year of transition to pass legislation in this arena (Haas, 2010). In 1994 the Chilean Government signed the Inter-American Convention on the Prevention, Punishment, and Elimination of Violence against Women. This increased the pressure of feminist activists on the Chilean Government to become active in this area, which Aylwin seemed open to, as he had already put stopping violence against women on the reform agenda. However, when the Socialist Party introduced a corresponding bill, the Senate called for cooperation with SERNAM (Haas, 2010). SERNAM converted the draft into a bill for the prevention and punishment of “family violence” to protect the family (and the woman in it with her “natural” role) (Ríos Tobar, 2007). Right-wing parties were bothered by the term “family violence”, since it questioned the natural order of the institution of marriage (Hiner and Azocar, 2015). Aylwin solved the dilemma, once again, by founding a National Family Commission. As in the case of the Rettig Commission, the aim was to establish a consensus and an expert committee. Aylwin stated that he wanted to avoid “to go to the press, get involved in controversies or produce spectacular effects.” He continued, “[I]t is a commission like the Rettig Commission, which accomplished its mission well, that aims to work in silence and with due gravitas” (speech by Patricio Aylwin cited in Hiner and Azocar, 2015, p. 62).

When the bill finally entered the conservative Senate in 1993, the debate on family violence was framed in antifeminist terms: “[T]here is no worse family violence than abortion and divorce,” noted Senator Eugenio Cantuarias, member of the right-wing party Unión Demócrata Independiente. The senator of the ruling Christian Democratic Party, Nicolas Díaz, expressed his opinion, which still influences the discussion on women's and reproductive rights: “[T]he most brutal violence is that used to assassinate a child in the uterus” (cited in Hiner and Azocar, 2015, p. 62–63). From an initially feminist reform proposal emerged a discussion that granted antifeminist discourse a prominent place.

### The Case of Matrimonial Law and the Right to Divorce

The right to divorce is another area in which there have been long-standing legislative initiatives and in which rights have been enforced substantially late.

In 1910, María Esíndola Núnez, the Chilean delegate of the Int'american Federation, informed that women had won the dispute on divorce in both New Zealand and Uruguay. It was not until 1958 that Inés Enríquez, the first female member of parliament, proposed a divorce bill. In the new family status of Allende from 1971, it was also planned to set up family tribunals to facilitate the divorce (Vitale n.d.). It took until 2004, with the law on civil marriage that divorces were made possible. Chile was the last country in Latin America to introduce divorce law. In practice, however, it has often been circumvented by a simulated action for annulment; for this “divorce á la chilena,” the spouses' assertion, confirmed by two witnesses, that at the time of the marriage they did not live in the district of the civil servant in charge of marriages was necessary (Gómez Urrutia, 2012).

The lack of divorce law was based on the continuation of a traditional, patrimonial family image, as in the case of barriers to enforce a law on domestic violence. In the constitution, which was adopted under Pinochet in 1980, the ideas of Allende were no longer represented. There was no definition of family there either. The benefit of missing a definition was that it did not take into account the variety of family forms that existed in the history of Chile. Instead, the image of the nuclear family of man and woman could be implicitly accepted (Gómez Urrutia, 2012). Until the modification of the Código Civil the woman had the role of an incompetent legal entity. Her property rights automatically passed to the man with the marriage. The introduction of a divorce law would have endangered this patrimonial family image: The indissolubility of marriage was perceived as the last legal bastion for the traditional family. By important political sectors it was therefore considered as an expression of the values that give rise to an orderly and stable society (Gómez Urrutia, 2012, p. 188 translated by the author^4^). As in the case of reproductive rights, it was argued that legal reform puts the family at risk as the core of national unity. And even in the case of divorce law, the Catholic Church took active position against divorce (Gómez Urrutia, 2012). Since the church was closely linked to the Concertación^5^, it slowed down the reactions of the parties of the Concertación regarding the activists‘demands (Guzmán et al., 2010). The church also contributed significantly to the fact that, in parallel with the case of reproductive rights, a veritable demonization of divorce law took place in the Senate and Congress debates. Gómez Urrutia (2012), who examined the debates on divorce law during the Concertación, found that the right to divorce was framed as being socially disintegrating. There were fears that poverty in general and drug use and juvenile crime would increase if divorce law would be introduced. (Gómez Urrutia, 2012). The following excerpt of a speech of a deputy illustrates the fears associated with the divorce law: “For the society it matters whether the family is stable or not; [society] cannot give up its right to promote the values on which it is founded, and the family is the most important of all. Therefore, the law must protect and promote the stability of marriage […] In consequence, it cannot be stated that divorce does not threaten the stability of the family and therefore the whole society” (Male deputy, Center Right party (PDC, cited in Gómez Urrutia, 2012, p. 189, translated by the author^6^).

In conclusion, both in the case of divorce law and in the case of domestic violence, it can be summarized that actor constellations and related discourses ensured that, despite the political opening up in Chile since the mid-1990's, the project of legal reforms in women's rights was postponed for 14 years. In the case of divorce law, it was not until 2004 that a change in the law of civil marriage brought about a reform that Guzmán et al. (2010) would classify as rather restrictive.

### The Role of Sernam

What role did SERNAM as the institution in charge of women and gender policy play? And what role did the women's movements play in relation to SERNAM?

SERNAM assumed a moderate position in this dispute to avoid damaging its own institution. Controversial issues such as domestic violence and reproductive rights were downplayed and less controversial issues of gender equality were taken up.

Rather than strengthening women's rights in the family, SERNAM sought political support by focusing on children's rights. According to Josefina Bilbao, who was an independent MP affiliated to the Christian Party of Chile and from 1994 to 2000 Minister of SERNAM, this was for strategic reasons. Thus, the impression should be avoided that the SERNAM pursued a distancing from the traditional family picture (cited in Gómez Urrutia, 2012).

Differences—deviant gender images or identities (e.g., indigenous groups such as the Mapuche^7^)—had no room in this conception of gender policy; the intersectionality of discrimination on the basis of race and sex was therefore neglected by SERNAM (Richards, 2005). An activist who identifies as Mapuche describes her interaction with SERNAM concerning the acceptance of intersectionality in politics in the following passage: “It hasn't been talked about [intersectionality], this is only starting recently. But generally before, they talked about the issue of gender, and that doesn't fit, because the relations between men and women in Western culture is one, and within Mapuche culture, it's another. Our way of relating with men is different. So long as they don't recognize us as a people, they are always going to try to assimilate us, so that we will be the same as the Chileans” (Richards, 2005, p. 212).

### The Role of the Women'S Movement

Considering the broad landscape of feminist organizations and women's movements that had contributed decisively to ending the Pinochet dictatorship, how was such a normalization of antifeminist and conservative gender models possible at all? First, it must be noted that social movements generally lost their influence in the transition to democracy, as political participation—similar to the time before the dictatorship—was concentrated strongly on the political parties. If the “public invisibility” (Chuchryk, 1994) of women had opened a window of opportunity for political organization during the dictatorship, it now limited their engagement: the candidates of the first elections were mostly male. This is also the fault of the binomial electoral system^8^, which is advantageous for well-known politicians from established families and disadvantageous for less-familiar female candidates.

For this reason, a group of women belonging to the parties of the center-left coalition came together with independent feminists in the Concertación de Mujeres por la Democracia (Coalition of Women for Democracy). They formulated a program to be included in the election program Concertación (Chuchryk, 1994). However, this led to further divisions between the women's movements: The presence of a common enemy, Pinochet, had bonded the extremely heterogeneous women's movements. Many leftist activists had already established close ties with the Left and Center parties during the dictatorship. This close connection to the state provoked protest among many smaller radical organizations and led to the division of the movements. This division was encouraged by the institutionalization of women's policy in expert committees and institutions, such as the SERNAM, and the establishment of a state feminism in which, most importantly, the “institutionalists” (Forstenzer, 2017, p. 171) among the activists were included. Their strategy was to adapt feminist demands to the dominant power relations and social narratives. In addition to the institutionalists and radical activists, there was another group of young women who, after the end of the dictatorship, focused their efforts on organizations that reflected traditional gender roles, e.g., parent organizations and religious associations (Marques-Pereira, 2005). In general women were only rarely political party members or worked actively in political parties. And Marques-Pereira noted the political disinterest of young Chileans during the transition period, which was evident in their low rates of electoral registration, i.e., a large portion of young Chileans passed up their chance to vote.

To sum up, during the transition to democracy, Chilean gender policies, as well as the treatment of dictatorship violence, focused on generating a politics of consensus in expert committees. As a result, everyday violence and oppression in Chilean society remained in the dark: “After so many years of violent dictatorship, we as a society were inclined to blame the military and thus were unable to recognize the daily violence in our families” (Bacigalupe, 2000, p. 438).

A hegemonic discourse became in due time the norm. This discourse determined societal relations and the way to deal with the authoritarian past: through the narrative of reconstruction, Chilean society was depicted as a whole. Key to this discourse was the hegemonic unity of the Chilean people, which excluded deviant gender images or identities. This normalization was made possible by a division of civil society in view of its relation to the transition state. The price paid for the institutionalization of gender policies and for allowing female activists to hold political positions was an extreme narrowing of women's and gender policies to the dominant narrative. Thus, parts of the gender regime were democratized, the parts that had access to political power and resources. But in concrete policies—in the underlying mentalities and attitudes of those who took part in shaping these policies—there was also a normalization of aspects of the authoritarian gender regime.

## Discussion and Conclusion

In the transition to democracy, the conservative gender images and roles that emerged during the military dictatorship or even earlier were perpetuated. As has been shown, more than two decades after the end of the dictatorship, these conservative discourses still limit the political frame and possibilities. This is also due to divided attitudes toward gender equality and the gendered division of labor in society. A survey by the United Nations Development Program (UNDP) shows that while 62 per cent of the population strongly support or at least accept the traditional role distribution (UNDP, 2010, p. 16), 38 per cent of the population, especially young adults and women, at least support the equal distribution of roles or believe that the state should be more committed to gender equality and diversity.

How this perpetuation of conservative gender images and roles limits reform ambitions can be seen in the two presidencies of Michelle Bachelet, who explicitly sought gender and social policy reform but only could show mixed results. The institutionalization of SERNAM, the introduction of a legislation to decriminalize therapeutic abortion and the change of the binomimal electoral system are clear milestones of Bachelet's presidencies (2006–2010 and 2014–2018) (Staab, 2016; Thomas, 2016) On the other hand, Bachelet had to bring in all her presidential power to get these reforms passed. Especially the case of social reforms shows that only compromises were possible that left the picture of the caring mother intact. On example is the struggle for paid care work. Bachelet was seeking for a general pay of unpaid work but due to protest, the compromise was a maternalist policy exclusively for women: women now receive child “bonuses” for their unpaid care work (Staab, 2016). By not recognizing the importance of gender equality in care work, the traditional image of the caring mother was strengthened (Staab, 2017). Therefore, the Chilean case shows that traditional gender roles are extremely persistent in policy making.

What general conclusions can be drawn from this case study of the interplay between liberalization and authoritarian gender discourses?

First of all, it shows that dealing with the authoritarian past in democratization processes causes high costs that can negatively affect the liberalization of gender relations. In the Chilean case a discourse of reconciliation was established, which conceived of Chileans as a unified whole in an effort to prioritize (economic) progress. This kind of reconciliation policy can be problematic as they have an influence on the room for divergent roles, gender images, and conflictive topics. In the Chilean case attempts to implement international standards on the national level were unsuccessful, because they were reinterpreted and integrated into conservative notions of gender relations. This is also a contradiction to the literature of international norm creation, which assumes that national norm entrepreneurs can achieve national liberal advances by recourse to the international level. A general learning is that despite international links of actors international norms may have small impact on authoritarian processes if they are not bound to sanctions.

A second general finding aims at the institutionalization of gender relations. The Chilean example shows that the emergence of state feminism and the integration of “institutionalists” within the women's movement into the National Women's Secretariat SERNAM led to a split between radical women's movements and institutionalists. The establishment of a hegemonic discourse was possible by establishing a state feminism and integrating the “institutionalists” among the women's movements into SERNAM. This integration divided radical women's movements and institutionalists. Such an institutionalization and cooptation can weaken feminist demands, as the coopted groups have to “pay” for its access to power by adapting feminist demands to the dominant power relations and societal narratives. Therefore, despite of state feminism, authoritarian gender regime may only undergo partial reforms. Formal access to positions of power and resources may change, but the conservative discourses and gender images tend to be carried over to democratic regimes.

A third finding is revealed by the comparison of the policy cases. The reform processes differ in duration and role of international norm building. However, in both cases we observe that the linkage of gender issues and family issues supported conservative norm building. The family was seen as the nucleus of society. Both policy reforms were perceived by conservative actors, especially the Catholic Church, as endangering this nucleus.

In the case of divorce law, a link of the reform project to social change in general increased the conservative backlash. Divorce was framed by conservative actors as leading to juvenile crime and social disintegration.

This leads to a last finding regarding the current phase of Chilean gender politics. It has been shown that authoritarian gender regimes can be of long duration, as the recent cases of sexual violence show. This is also due to the interaction with other authoritarian institutional enclaves, such as the electoral system. In the case of Chile, before the reforms of Bachelet, the previous electoral system brought about a narrowing of political content, since candidates representing critical issues did not receive a place on the party lists. The literature on gender effects of electoral reforms shows, however, that even reforms of electoral systems that have gender equality as a perspective, do not necessarily lead to a higher substantive representation. However, as Waylen (2006) shows in the example of the constitutionalization process in Iraq^9^, the guarantee of descriptive representation is not enough if in the transition process there are already elements of an authoritarian gender regime in the constitution.

## Author's Note

Parts of the theoretical framework and the empirical part on reproductive rights are already published in Graf, Patricia 2018: In the Shadow of Autocracy. Gender Politics in Chile. In: Wilde, Gabriele/Zimmer, Annette/Obuch, Katharina/Sandhaus Jasmin: Civil Society and Gender Relations in Authoritarian and Hybrid Regimes.

## Author Contributions

The author confirms being the sole contributor of this work and has approved it for publication.

### Conflict of Interest

The author declares that the research was conducted in the absence of any commercial or financial relationships that could be construed as a potential conflict of interest.

## Footnotes

^1^However, it ranks better than Mexico (Rank 76), Brazil (Rank 94), and Argentine (Rank 81).

^2^In democratic as well as authoritarian gender regimes, the resources and power positions can be unequally distributed between the genders. In authoritarian gender regimes, however, there is a lack of control by accountable institutions and actors.

^3^The Chilean constitution does not allow a direct reelection; the term of office is four years.

^4^“Vista como el último bastión legal para la familia tradicional, la indisolubilidad del matrimonio era considerada por importantes sectores políticos como expresión de los valores que dan origen a una sociedad ordenada y estable.”

^5^The “Concertación por la Democrácia” was the name for a Coalition of center-left parties. The strongest party in this coalition was the Christian Democratic Party (PDC). It was in power between 1990 and 2010.

^6^“A la sociedad no le da lo mismo que la familia sea estable o no; ella no puede renunciar a su derecho a promover los valores en que se funda, y la familia es el más importante de todos. Por eso, la ley debe proteger y fomentar la estabilidad del matrimonio […] En consecuencia, no se puede decir que el divorcio no atenta contra la estabilidad de la familia y, por ende, contra la sociedad en su conjunto”.

^7^The Mapuche are an indigenous people, mainly located in the capital region, the 8th and 9th regions of Chile, as well as in Argentina.

^8^On the party lists, the number of female and male candidates must not exceed 60 per cent of the total candidates. For every female candidate, the party will be “rewarded” by the state. Parties receive $20,000 for each elected female representative or senator.

^9^“Article 14 states that Iraqis are equal before the law ‘without discrimination because of sex' at the same time as it states that laws cannot be passed that contradict the ‘established rulings' of Islam” (2006, 1218).
